# The relationship between body dissatisfaction and attentional bias to thin bodies in Malaysian Chinese and White Australian women: a dot probe study

**DOI:** 10.1098/rsos.230674

**Published:** 2023-09-20

**Authors:** T. House, H. K. Wong, N. W. Samuel, I. D. Stephen, K. R. Brooks, H. Bould, A. S. Attwood, I. S. Penton-Voak

**Affiliations:** ^1^ School of Psychological Sciences, Faculty of Medicine, Health and Human Sciences, Macquarie University, Australia; ^2^ School of Psychological Science, University of Bristol, UK; ^3^ School of Psychology, University of Nottingham Malaysia, Malaysia; ^4^ NTU Psychology, Nottingham Trent University, Nottingham, UK; ^5^ Perception in Action Research Centre (PARC), Macquarie University, Australia; ^6^ Lifespan Health and Wellbeing Research Centre, Macquarie University, Australia; ^7^ Centre for Academic Mental Health, Population Health Sciences, Bristol Medical School, University of Bristol, UK; ^8^ Gloucestershire Health and Care NHS Foundation Trust, Gloucester, UK; ^9^ MRC Integrative Epidemiology Unit, Bristol Medical School, University of Bristol, UK; ^10^ National Institute for Health Research Bristol Biomedical Research Centre, University Hospitals Bristol NHS Foundation Trust and University of Bristol, UK

**Keywords:** attention, attentional bias, body dissatisfaction, body size, dot probe

## Abstract

Studies suggest that an attentional bias to thin bodies is common among those with high levels of body dissatisfaction, which is a risk factor for, and symptom of, various eating disorders. However, these studies have predominantly been conducted in Western countries with body stimuli involving images of White people. In a preregistered study, we recruited 150 Malaysian Chinese women and 150 White Australian women for a study using standardized images of East Asian and White Australian bodies. To measure attentional bias to thin bodies, participants completed a dot probe task which presented images of women who self-identified their ethnicity as East Asian or as White Australian. Contrary to previous findings, we found no evidence for an association between body dissatisfaction and attentional bias to thin bodies. This lack of association was not affected by participant ethnicity (Malaysian Chinese versus White Australian) or ethnic congruency between participants and body stimuli (own-ethnicity versus other-ethnicity). However, the internal consistency of the dot probe task was poor. These results suggest that either the relationship between body dissatisfaction and attentional bias to thin bodies is not robust, or the dot probe task may not be a reliable measure of attentional bias to body size.

## 1. Introduction

Body dissatisfaction is defined as the negative subjective evaluation of one's body and is often considered the attitudinal manifestation of body image disturbance [1]. Body dissatisfaction is a risk factor [2] and symptom [3] of eating disorders, such as anorexia nervosa, making it a potential target for therapeutic intervention. High levels of body dissatisfaction are associated with multiple appearance-related attentional biases [4]. For example, eye-tracking studies show women with high (compared to low) body dissatisfaction spend more time looking at self-defined unattractive body parts when presented with their own body and self-defined attractive body parts when presented with another person's body [5,6]. Further, eye-tracking studies presenting women with images of other people consistently show that women reporting high (compared to low) levels of body dissatisfaction, spend more time fixating on thin women [7–11]. This association can be explained by the tripartite model of body image, which suggests that sociocultural pressures lead women to internalize the thin-ideal and compare their body to others, and as a result women feel less satisfied with their own body [12]. Sociocultural pressure (from, for example, Western media) has a long history of presenting thinness as aspirational for women [13–17]. The thin-ideal is reflected in women's body size preferences: women consistently rate thinner bodies as more attractive [18,19].

The effects of appearance comparisons can be further explained by social comparison theory, which states that people evaluate themselves by making upward social comparisons to people they perceive as more attractive and downward social comparisons to people they perceive as less attractive [20,21]. Upward comparisons are proposed to increase negative emotions, whereas downward comparisons are proposed to increase positive emotions. In support of this, ecological momentary assessment studies have found upward social comparisons to be associated with increased body and appearance dissatisfaction [22,23]. Further support comes from experimental research showing that viewing thin bodies can lead to increased body dissatisfaction [24–27], particularly among people at risk of developing an eating disorder [28,29]. Therefore, attentional bias to thin bodies may exacerbate body dissatisfaction in women.

While eye-tracking studies support the positive association between body dissatisfaction and attentional bias to thin bodies [7–11], evidence is less consistent when the dot probe task is used to measure attentional bias. The dot probe task presents participants simultaneously with a target stimulus (e.g. a thin body) alongside a control stimulus (e.g. a non-thin body or a non-body object). Participants respond to a probe replacing one of the stimuli, and faster reaction times to probes replacing target stimuli compared to control stimuli are interpreted as an attentional bias towards target stimuli [30]. Some dot probe studies have found support for a positive association between body dissatisfaction and attentional bias to thin bodies [31–33], whereas other studies found no such evidence [33–35]. However, findings from these studies are potentially limited by their small samples sizes [33,34] and reduced number of dot probe trials [35]. Further, many of the dot probe tasks used a stimulus-onset asynchrony (SOA; the interval between the onset of the stimulus pair and the onset of the probe) of greater than or equal to 500 ms [31,32]. Chapman *et al.* [36] found that shorter SOAs (less than 300 ms) improved the reliability of the dot probe task, possibly because participants had less time to redistribute their attention before responding to the probe. However, evaluation of the reliability of dot probe studies is made difficult by the general lack of reporting on the psychometric properties of cognitive-behavioural tasks [37].

Another common feature of the discussed dot probe studies [31–35] is that they all presented White body stimuli to people in Western countries. Although body image disturbance was once considered culturally bound to Western societies, the globalization of Western media is thought to have contributed to body dissatisfaction and adoption of the thin-ideal in many non-Western countries [18,38]. This is particularly relevant in Malaysia, a newly industrialized country in South East Asia where recent findings suggest over 50% of adults experience eating disorder symptoms [39]. Body image disturbance is common in Malaysia—prevalence studies estimate that 48.1% of undergraduate women want to be thinner [40] and 88% of female adolescents have body shape concerns [41]. Cross-cultural body image research highlights some commonalities between Malaysian and Western populations; however, findings are somewhat piecemeal. People in urban areas of Malaysia reported a similar preference for low body mass index (BMI) bodies as people in Britain, while people in rural areas of Malaysia preferred higher BMI bodies [42]. In one study, Malaysian Chinese women from urban areas of Malaysia reported greater body dissatisfaction than Australian women [43]. In another study, Australian women reported higher body dissatisfaction than Malaysian women, although effect sizes were very small [44]. Shagar and colleagues tested the tripartite model of body image in Australian and Malaysian women. Although there were some differences between populations, the theoretical framework of the tripartite model of body image could be applied similarly to both [45].

In the present study, we used a dot probe task to examine the association between body dissatisfaction and attentional bias to thin bodies. We recruited a sample of Western (White Australian) and non-Western (Malaysian Chinese) women and presented them with both White Australian and East Asian body stimuli. To overcome limitations from previous dot probe research, we recruited a relatively large sample size with enough statistical power to detect an association separately in both populations of women. We also used a relatively high number of trials for the dot probe task. Based on the findings of Chapman *et al.* [36], we aimed to increase the reliability of the dot probe task by using a short SOA (100 ms). We also evaluated the reliability of the dot probe task by estimating the task's internal consistency. We hypothesized that body dissatisfaction would be positively associated with attentional bias towards thin bodies, so women with higher body dissatisfaction would have a greater attentional bias towards thin bodies. We also explored the moderating role of participant ethnicity (White Australian versus Malaysian Chinese) and the ethnic congruence between participants and body stimuli (own-ethnicity versus other-ethnicity). The study protocol was preregistered on the Open Science Framework (https://osf.io/yt5fh/) with variations from the protocol explained in electronic supplementary material.

## 2. Material and methods

### 2.1. Participants and recruitment

We aimed to recruit 150 Malaysian Chinese and 150 White Australian participants, giving over 90% power to detect an effect size of *r* = 0.26 in each group (we reduced the effect size reported by Dondzilo and colleagues by 33% to account for the inflation of published effect sizes [31,46]). Two Malaysian Chinese participants and one White Australian participant responded correctly on fewer than 60% of the dot probe trials. We excluded these participants and recruited replacement participants to reach our target sample size for each group. Participants were required to be 18–35 years old, female, and either White Australian (Australian sample) or Malaysian Chinese (Malaysian sample). Participants were not screened for current or past eating disorder diagnosis. White Australian participants were recruited via Macquarie University's study signup system and reimbursed with course credit. For the Malaysian Chinese sample, 83 participants were recruited via University of Nottingham Malaysia's study signup system (reimbursed with course credit) and 67 participants were recruited via social media adverts and snowball sampling (reimbursed with RM5 (approx. US $1.20)).

### 2.2. Measures

#### 2.2.1. Demographics

To ensure participants met our eligibility criteria, we used a demographics questionnaire (see electronic supplementary material) that asked participants to report their ethnicity, gender and age in years. The questionnaire also asked participants to report their height and weight, so that we could calculate their BMI (kg m^−2^).

#### 2.2.2. Body dissatisfaction

Body dissatisfaction was measured using a modified version of the Body Shape Satisfaction Scale [47]. This version of the questionnaire asked participants to rate their satisfaction with 16 parts or features of their body (e.g. waist, hips and thighs) using a Likert scale ranging from 1 to 7 (1 representing ‘Very dissatisfied’ and 7 representing ‘Very satisfied’; see electronic supplementary material). Responses for each item were reverse scored and a single body dissatisfaction score calculated for each participant by summing responses for all items. Scores could range from 16 to 112, with higher scores representing greater body dissatisfaction. The questionnaire was originally developed in the English language and we presented it in English for both White Australian and Malaysian Chinese participants. English is widely spoken in Malaysia as a second language [48] and in most universities is the primary language of instruction. The majority of Malaysian Chinese participants were studying at a British branch university campus where overall English proficiency level is high (e.g. for undergraduate studies, the university requires a minimum score of 6.0 in the International English Language Testing System (IELTS) or equivalent). The questionnaire was also evaluated for appropriateness to local contexts by authors H.K.W. and N.W.S. who are Malaysian Chinese and multilingual, speaking English, Mandarin and Malay proficiently. The 16 item version of the questionnaire has shown high internal consistency and convergent validity in studies on Australian women [49–51]. An earlier 10 item version of the questionnaire has also demonstrated test–retest reliability, and concurrent and predictive validity in female adolescents in the United States [52–54]. In our sample, Cronbach's alpha for the scale demonstrated excellent internal consistency for both Malaysian Chinese women (*α* = 0.94) and White Australian women (*α* = 0.91).

#### 2.2.3. Stimuli

**Figure 1.**
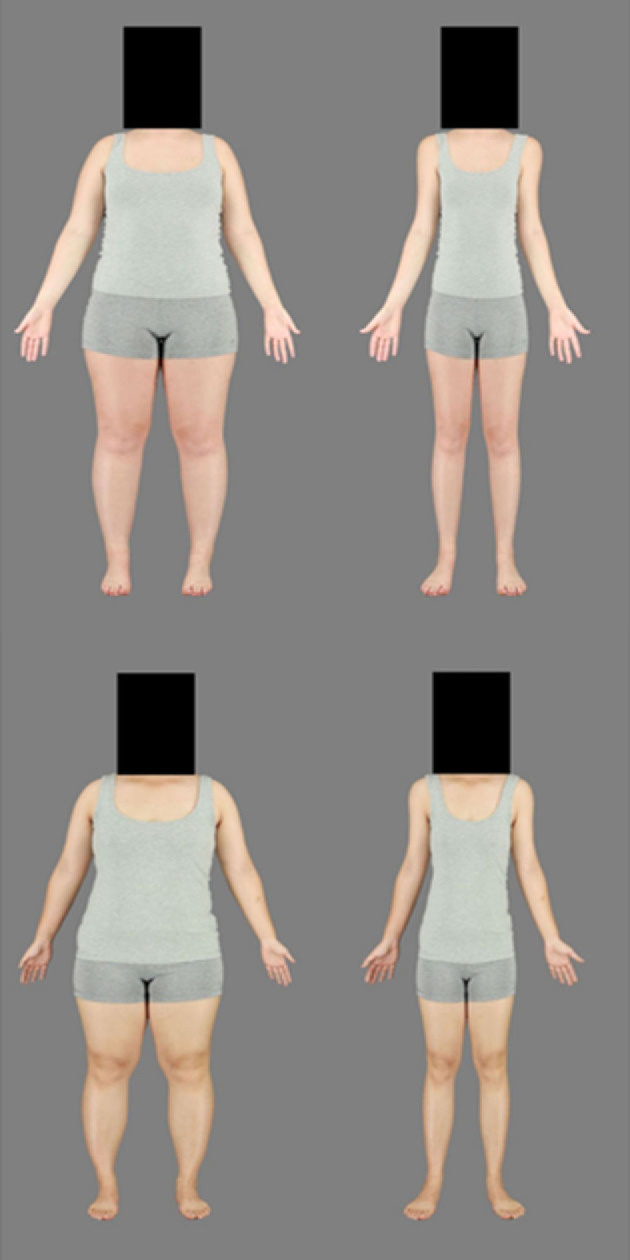
Example body stimuli depicting expanded (left) and contracted (right) versions of the same identities. Body stimuli on the top row are of a woman identifying as White Australian, while those on the bottom row are of a woman identifying as East Asian.

Body stimuli were obtained from previous research conducted on women recruited in Australia who identified as East Asian or White Australian and provided written consent for their photographs to be used in future research [55]. The photographs were taken using standardized angle and lighting conditions and the women stood in the same anatomical pose wearing the same grey singlet and shorts. Body stimuli selected for the present study consisted of 10 East Asian identities and 10 White Australian identities, matched for BMI. For each identity, the Spherize tool in Photoshop was used to create versions simulating higher and lower BMIs [55]. This involved horizontal expansion or compression, respectively, which was maximal (50%) around the navel, but diminished towards the neck and ankles. This approach for simulating BMI differences has been effectively used in previous research on body size perception [55–57]. All body stimuli had faces covered with a black square to prevent any influence of facial characteristics (figure 1). We defined the body stimuli based on the congruence between stimulus ethnicity and participant ethnicity, so own-ethnicity body stimuli involved East Asian stimuli presented to Malaysian Chinese participants and White Australian stimuli presented to White Australian participants. Other-ethnicity body stimuli involved East Asian stimuli presented to White Australian participants and White Australian stimuli presented to Malaysian Chinese participants.

#### 2.2.4. Dot probe task

**Figure 2.**
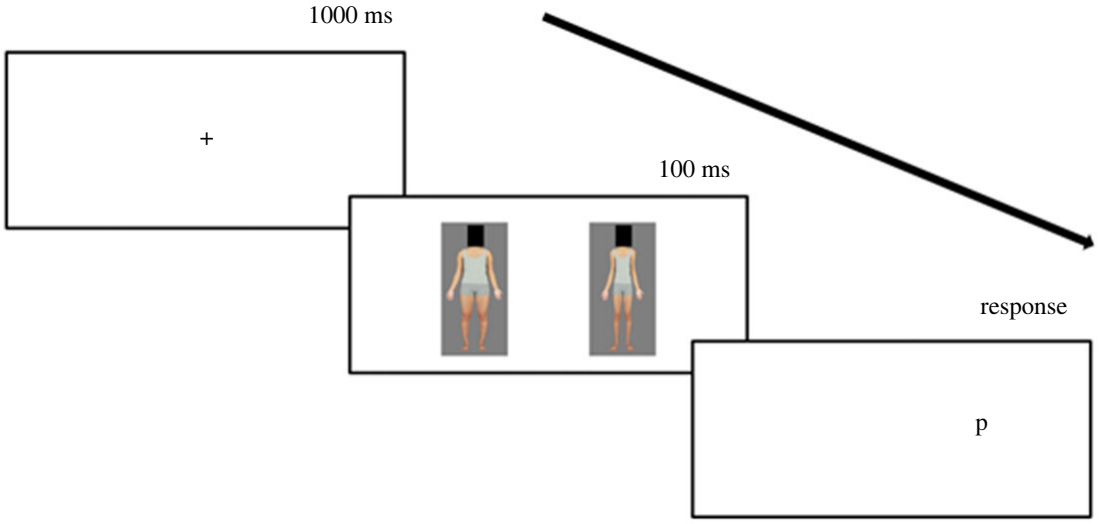
Example dot probe trial where the body stimuli involved an expanded and a contracted version of the same East Asian woman. In this example, the probe (letter ‘p’) replaced the contracted target body.

Attentional bias was measured using a modified dot probe task [58]. Each trial started with a 1000 ms presentation of a fixation cross, followed by two body stimuli (one expanded and one contracted version of the same identity) presented simultaneously for 100 ms on either side of the fixation cross (sides randomized; figure 2). The body stimuli then disappeared, and a probe appeared (either the letter ‘p’ or ‘q’) in the location previously occupied by one of the body stimuli. The probe location was randomized, having an equal probability of replacing each body stimulus. Participants were required to identify the letter as quickly and accurately as possible by pressing the corresponding keyboard button (either ‘p’ or ‘q’).

The dot probe task consisted of 320 trials divided into four blocks of 80, with a 30 s break between each block. Two blocks presented participants with own-ethnicity body stimuli while the other two presented participants with other-ethnicity body stimuli. The block order, and order of stimulus presentation within each block, was randomized for each participant. To compute attentional bias scores, we followed previous dot probe studies and excluded trials when the participant responded incorrectly or when their reaction time was less than 200 ms or more than 2.5 standard deviations above their mean reaction time [31]. Mean response times for the remaining trials were used to generate attentional bias scores using the following formula [58]:

$$\mathrm{Attentional}\;\mathrm{bias}\;\mathrm{score}=\frac{\left(\left[\mathrm{LPRT}\text{–}\mathrm{LPLT}]+[\mathrm{RPLT}\text{–}\mathrm{RPRT}\right]\right)}{2}.$$

Here, ‘L’ refers to the left side of the screen, ‘R’ refers to the right side of the screen, ‘P’ refers to the probe and ‘T’ refers to the target stimulus (which for this study was the contracted body stimulus). For example, ‘LPRT’ refers to the mean response time when the probe (P) was located on the left (L) but the contracted body stimulus target (T) was located on the right (R) and so on. A positive attentional bias score represents a bias to contracted body stimuli while a negative attentional bias score represents a bias to expanded body stimuli.

#### 2.2.5. Procedure

Participants provided informed consent and completed the study online via Gorilla (https://gorilla.sc/) [59]. The demographics questionnaire was completed first, followed by the Body Shape Satisfaction Scale, followed by 10 practice dot probe trials that were identical to the main dot probe trials, except that participants were presented with a green tick for responding correctly and a red cross for responding incorrectly. Body stimuli for the practice trials were selected at random from the pool of 20 identities. Participants then completed the main dot probe task, followed by a debrief.

#### 2.2.6. Data analysis

All analyses were conducted using R (v.4.2.1) [60]. We conducted preliminary analyses to assess group differences between Malaysian Chinese and White Australian participants for body dissatisfaction, age, BMI and attentional bias scores (separately for own-ethnicity and other-ethnicity body stimuli). Due to some variables being non-normally distributed, we assessed group differences using bootstrapped independent *t*-tests and the MKinfer R package [61]. Bootstrapped statistics were bias-corrected and accelerated, using 5000 iterations. We then conducted three preregistered linear mixed effects models using the lme4 R package [62]. Residuals demonstrated minor deviations from normal distributions; however, linear mixed effects models are generally robust to these deviations [63].

For model 1, we ran a random intercepts model using the restricted maximum-likelihood approach to predict attentional bias from the fixed effect of body dissatisfaction, including age and BMI as confounding fixed effects and participant ID as a random effect. We centred the variables body dissatisfaction, age and BMI using group mean centring separately for Malaysian Chinese and White Australian participants. We estimated *p*-values using the Satterthwaite's degrees of freedom method with the lmerTest R package [64] and inferred support for our hypothesis if body dissatisfaction had a positive coefficient (*p* < 0.05). For model 2, we explored the moderating role of participant ethnicity by dummy coding this variable (Malaysian Chinese = 0 and White Australian = 1) and adding it to model 1 as a fixed effect to interact with body dissatisfaction. We inferred evidence for a moderating role of participant ethnicity if there was an interaction between body dissatisfaction and participant ethnicity (*p* < 0.05). For model 3, we explored the moderating role of ethnic congruency by dummy coding this variable (other-ethnicity = 0 and own-ethnicity = 1) and adding it to model 2 as a fixed effect to interact with body dissatisfaction. We inferred evidence for a moderating role of ethnic congruency if there was an interaction between body dissatisfaction and ethnic congruency (*p* < 0.05). We aimed to explore significant interactions using follow-up simple slope analyses.

We conducted three additional exploratory analyses that were not preregistered. First, to further understand null results, we conducted Bayesian bivariate correlations to test the relationship between body dissatisfaction and attentional bias to contracted bodies. This was done separately for each participant group and ethnic congruency condition. Due to the non-normal distribution of some variables, we conducted Spearman's rank-order correlations. We calculated Bayes factors using the correlation R package [65] to determine the likelihood of the alternative hypotheses (*r* ≠ 0) in relation to the corresponding null hypotheses (*r* = 0). We interpreted Bayes factors using the JASP classification scheme, so Bayes factors greater than 1 would provide support for the alternative hypothesis and Bayes factors smaller than 1 would provide support for the null hypothesis [66].

Second, we explored the internal consistency of the dot probe task using the splithalf R package [67], which estimates split-half reliability statistics for cognitive tasks. To use the package, we coded dot probe trials as congruent when the contracted body stimulus appeared on the same side of the screen as the probe. We coded trials as incongruent when the contracted body stimulus appeared on the opposite side of the screen to the probe.^1^ We then used splithalf to calculate the average Spearman–Brown corrected correlation coefficients for 5000 random splits. We estimated reliability statistics separately for each participant group and ethnic congruency condition. Third, to test the robustness of our results, we conducted a sensitivity analysis and reran all analyses without outliers to assess whether the results were driven by extreme values. Following the approach of previous dot probe research, we defined outliers as values over 3 standard deviations above or below the mean [31].

## 3. Results

We excluded dot probe trials where participants responded incorrectly (4.39% of dot probe trials for Malaysian Chinese participants and 6.60% of dot probe trials for White Australian women). For remaining trials, we excluded trials when the participant's reaction time was less than 200 ms (0.05% of correct trials for Malaysian Chinese participants and 0.10% of correct trials for White Australian participants) or more than 2.5 standard deviations above the participant's mean reaction time (2.06% of correct trials for Malaysian Chinese participants and 2.25% of correct trials for White Australian participants). Participant characteristics are presented in table 1 alongside the results of the bootstrapped independent *t*-tests. The results of the preregistered linear mixed effects models are presented in table 2. Model 1 found no evidence for an association between body dissatisfaction and attentional bias to contracted bodies. Model 2 found no evidence for an interaction between body dissatisfaction and participant ethnicity on attentional bias to contracted bodies. Model 3 found no evidence for an interaction between body dissatisfaction and ethnic congruency on attentional bias to contracted bodies. As we found no evidence for moderating effects, we did not conduct follow-up simple slope analyses.

**Table 1.** The descriptive statistics for the participant characteristics. Bootstrapped independent *t*-tests were used to compare participants on each characteristic. Statistics were bias-corrected and accelerated and used 5000 iterations. *Note.* We have reported the median (Mdn) and interquartile range (IQR) due to the non-normal distribution of some variables.

	Malaysian Chinese (*N* = 150)		White Australian (*N* = 150)		*t*	*p*-value
	Mdn	IQR	Mdn	IQR		
age (years)	22.00	5.00	18.00	4.00	−3.41	<0.001
body mass index (BMI)	19.72	4.12	22.51	6.33	5.60	<0.001
body dissatisfaction	64.00	21.50	64.00	24.00	0.62	0.540
attentional bias score to own-ethnicity body stimuli	1.46	28.14	2.17	27.80	0.93	0.348
attentional bias score to other-ethnicity body stimuli	0.01	22.74	−0.67	27.52	−2.06	0.011

**Table 2.** The results of the three linear mixed effects models with the outcome variable as attentional bias score (*N* = 300). CI = confidence interval.

effect	model 1			model 2			model 3		
	*β*	95% CI	*p*-value	*β*	95% CI	*p*-value	*β*	95% CI	*p*-value
body dissatisfaction	0.06	−0.03, 0.15	0.169	0.09	−0.02, 0.21	0.118	0.11	−0.04, 0.25	0.140
age	−0.02	−0.10, 0.06	0.605	−0.02	−0.10, 0.06	0.605	−0.02	−0.11, 0.06	0.606
body mass index (BMI)	0.00	−0.09, 0.09	0.982	0.00	−0.09, 0.09	0.950	0.00	−0.09, 0.09	0.950
participant ethnicity	—	—	—	−0.09	−0.25, 0.07	0.281	−0.09	−0.25, 0.07	0.282
body dissatisfaction × participant ethnicity	—	—	—	−0.07	−0.23, 0.09	0.413	−0.07	−0.23, 0.09	0.414
ethnicity congruency	—	—	—	—	—	—	−0.01	−0.18, 0.15	0.861
body dissatisfaction × ethnic congruency	—	—	—	—	—	—	−0.03	−0.19, 0.13	0.736

The Bayesian correlation analyses found moderate support for the null hypothesis for each participant group and ethnic congruence condition (White Australian own-ethnicity trials: *r* = 0.01, BF_10_ = 0.19; White Australian other-ethnicity trials: *r* = 0.08, BF_10_ = 0.29; Malaysian Chinese other-ethnicity trials: *r* = −0.02, BF_10_ = 0.19). The only exception was for Malaysian Chinese own-ethnicity trials where the result supported the alternative hypothesis; however, this support was only weak (*r* = 0.18, BF_10_ = 1.77). In split-half reliability analyses, the dot probe task demonstrated poor internal consistency for Malaysian Chinese participants (own-ethnicity trials: Spearman–Brown coefficient = 0.01 [95% CI = −0.53, 0.49]; other-ethnicity trials: Spearman–Brown coefficient = 0.50 [95% CI = 0.01, 0.75]) and White Australian participants (own-ethnicity trials: Spearman–Brown coefficient = −0.23 [95% CI = −0.67, 0.17]; other-ethnicity trials: Spearman–Brown coefficient = −0.06 [95% CI = −0.36, 0.24]). Lastly, the removal of outlier participants (seven Malaysian Chinese participants and five White Australian participants) did not substantially change our results (see electronic supplementary material).

## 4. Discussion

The results of this study did not support our preregistered hypothesis. We found no evidence for an association between body dissatisfaction and attentional bias to thin bodies, as measured on a dot probe task. To the best of our knowledge, this is the first dot probe study to explore the association between body dissatisfaction and attentional bias to thin bodies in a non-Western population using non-White body stimuli. We did not find evidence for a moderating role of participant ethnicity (Malaysian Chinese versus White Australian) or ethnic congruency between participants and body stimuli (own-ethnicity versus other-ethnicity). The absence of association between body dissatisfaction and attentional bias to thin bodies contrasts with certain dot probe studies that report a positive association [31–33]. However, the results are consistent with other dot probe studies that found no evidence for an association [33–35].

One possible reason for not finding an association between body dissatisfaction and attentional bias is our use of a larger body as a control stimulus. It is possible that our expanded and contracted body stimuli may not have been visually contrasting enough to produce measurable differences in attention. On the other hand, there is some preliminary evidence indicating that body dissatisfaction is positively associated with attentional bias to larger bodies [4], and so a larger body control stimulus may have attracted attention away from the target thin body stimulus. In their dot probe task, Dondzilo *et al.* [31] used control stimuli that did not involve bodies, which may have meant their thin body stimuli were more likely to capture the attention of participants. However, other studies using larger bodies for control stimuli have also reported a positive association between body dissatisfaction and thin bodies. For example, Joseph *et al.* [32] used thin body stimuli with an estimated BMI of 18 kg m^−2^ and larger body control stimuli with an estimated BMI of 36 kg m^−2^. Moussally *et al.* [33] used thin body stimuli with an estimated BMI of 15.67 kg m^−2^ and larger body control stimuli with an estimated BMI of 30.63 kg m^−2^. Our method of body stimuli creation did not enable us to estimate stimulus BMI, but our body stimuli do appear to be of a comparable size to those used by Joseph *et al.* [32] and Moussally *et al.* [33]. Therefore, it appears unlikely that our results were caused by the larger body control stimulus detracting attention away from the target thin body stimulus, or by using target and control stimuli that are too visually similar. In fact, extreme body sizes may reduce validity. Glauert *et al.* [34] presented extremely thin body stimuli (estimated BMI = 11.7 kg m^−2^) alongside larger body control stimuli (estimated BMI = 30.4 kg m^−2^) and found no evidence for an association between body dissatisfaction and attentional bias to thin bodies. Researchers have proposed that the null findings reported by Glauert and colleagues may be due to the thin body stimuli being so emaciated that they did not attract as much attention due to low ecological validity [32]. Our thin body stimuli were less extreme than those used by Glauert and colleagues, and hence should have been effective in capturing attention. However, we recommend further experimental exploration using stimuli with varying degrees of body size.

Another possible explanation for our results is that participants completed the study online in a location of their choosing rather than in a controlled laboratory setting, and may have experienced reduced motivation and more distractions. Dot probe studies reporting positive associations between body dissatisfaction and attentional bias to thin bodies were all conducted in a laboratory setting as opposed to online [31–33]. Therefore, a laboratory setting may be necessary to detect this positive association. However, other dot probe studies conducted in a laboratory setting failed to find evidence for an association [33–35], and one study found similar results regardless of whether the study was completed online or in a laboratory setting [35]. Therefore, a laboratory setting is certainly not a sufficient condition for detecting a positive association. We also excluded participants with poor dot probe accuracy, so we can assume participants were directing an acceptable level of attention to the task. It, therefore, appears unlikely that these inconsistent results are due to the study setting.

Another variable feature of dot probe studies is the SOA of the dot probe task, which refers to the interval between the onset of the stimulus pair and the onset of the probe. Dot probe studies reporting a positive association all used a 500 ms SOA [31–33], whereas studies using longer SOAs (1500 ms [33]) and shorter SOAs (100 ms [35] and 150 ms [33,34]) have not found evidence for an association. Therefore, the association may only be detectable using a 500 ms SOA. However, some studies using a 500 ms SOA failed to find evidence for an association [34,35] and a recent meta-analysis found no evidence for moderating effects of SOA on the association between body dissatisfaction and attentional bias to thin bodies [68]. Therefore, the association appears unlikely to be dependent on SOA; however, experimental studies are needed to confirm this. Chapman *et al.* [36] found that shorter SOAs (100 ms) improved the reliability of the dot probe task, possibly because participants had less time to redistribute their attention before responding to the probe. We aimed to increase the reliability of our dot probe task by using a similarly short SOA of 100 ms. However, this manipulation was clearly insufficient because our dot probe task still demonstrated poor internal consistency (*r* ≤ 0.50).

There is not a standard practice in psychological science for consistent reporting on the psychometric properties of cognitive-behavioural tasks [37]. Therefore, it is difficult to compare the reliability of our dot probe task to the other previously mentioned studies that measured attentional bias to body size. However, the low reliability of our dot probe task is consistent with other studies that have analysed the reliability of the dot probe task [36,69,70]. In fact, low reliability affects many other similar cognitive tasks used for individual difference research that calculate reaction times difference scores (e.g. the Stroop task) [71]. The poor reliability of reaction time tasks like the dot probe task and Stroop task may be explained by their reduced sensitivity as measures of attentional bias, because the tasks are unable to differentiate between reaction time differences caused by biased attentional engagement versus disengagement. For example, in the dot probe task participants may be faster at responding to probes replacing thin bodies due to fast engagement with the thin body or because once engaged with the thin body they are slow to disengage [72]. Alternative reaction time tasks like the ARDPEI task [73] and visual search task [74] may be able to overcome this limitation; however, to the best of our knowledge the psychometric properties of these tasks are yet to be evaluated for measuring attentional bias to thin bodies. Further, all aforementioned reaction time tasks have traditionally measured attentional bias using reaction time difference scores, which may be unreliable for measuring individual differences in attentional bias because they have low between-participant variability [71], do not capture the dynamic nature of attention over repeated trials [75], and rely on keyboard presses that are affected by variations in participant motor speed [76].

Although dot probe studies have produced inconsistent results, eye-tracking studies consistently show that women reporting high levels of body dissatisfaction, in comparison to women with low levels of body dissatisfaction, spend more time fixating on thin women [68]. Fixation durations are likely to produce more reliable estimates of attention when compared to reaction time difference scores on the dot probe task, because they do not rely on motor responses or aggregated scores [75,76]. Further, fixation durations measure attentional bias across the total stimulus presentation period rather than at one specific time point. Indeed, eye-tracking studies using indices such as total fixation duration report much higher reliability than dot probe measures of attention [77,78], which might explain why eye-tracking studies produce more consistent evidence for a positive association between body dissatisfaction and attentional bias to thin bodies. Support for this comes from research showing that eye-tracking and dot probe indices are generally not correlated despite both being common measures of attentional bias [78].

Given the poor reliability of our dot probe task, we do not think our results can be used with confidence to evaluate the association between body dissatisfaction and attentional bias to thin bodies. Eye-tracking research provides evidence for a positive association [7,8,10,11], including with a similar sample of White Australian women [9]; therefore, we think it is likely that the dot probe task was too unreliable to detect this association. To the best of our knowledge, no eye-tracking research has assessed body size attentional biases in Malaysian Chinese women. Therefore, we are unsure whether an association is absent in this population or whether we failed to detect an association due to the low reliability of the dot probe task. We did not find evidence for a moderating effect of participant ethnicity on the association between body dissatisfaction and attentional bias to thin bodies. However, given the poor reliability of the dot probe task we are cautious to eliminate the possibility of cross-cultural differences. There was some evidence indicating that White Australian women had a reduced attentional bias to thin bodies compared to Malaysian Chinese women (table 1). However, the comparison was exploratory and evidence for the difference was weak (*p* > 0.01); therefore, further research should continue to explore this comparison. Research indicates the tripartite model of body image can be applied similarly to Australian and Malaysian women [45]; however, we think eye-tracking research is needed to confirm the association between body dissatisfaction and attentional bias to thin bodies. Similarly, we did not find evidence for a moderating effect of the ethnic congruence of the body stimuli; however, more reliable measures of attentional bias may find such evidence.

### 4.1. Strengths and limitations

Strengths of this study include the sufficiently powered sample size, relatively high number of dot probe trials and preregistered study protocol. However, there are a number of limitations. First, we used the same body dissatisfaction questionnaire for both White Australian and Malaysian Chinese populations; however, to the best of our knowledge the questionnaire has not had its psychometric properties assessed in a Malaysian population. We chose this questionnaire to increase comparability between populations; however, we cannot be certain that body dissatisfaction can be defined and measured equally between different cultures [79]. The questionnaire did not require translation because it was presented to an English-speaking population. Further, the questionnaire is relatively simple and was evaluated for appropriateness to local contexts by authors H.K.W. and N.W.S. who are Malaysian Chinese and multilingual, speaking English, Mandarin and Malay proficiently. A variation of the questionnaire has been shown to correlate with eating disorder symptoms in a similar Malaysian population (undergraduate students from Kuala Lumpur and Selangor, Malaysia) [80]. Therefore, it seems likely that our questionnaire is valid in this population, although further research is required to confirm this.

Second, to assess body stimulus ethnic congruence (own-ethnicity versus other-ethnicity) we presented participants with body stimuli depicting women identifying as White Australian or East Asian. However, the ethnic congruence of the stimuli may not have been equivalent for each participant group. Third, although our stimulus transforms were relatively effective in simulating increases or decreases in BMI, more sophisticated fat transforms may be used to achieve more realistic differences [81]. The body stimuli should also have been rated separately in a pilot study to be sure they were equated for key characteristics, such as perceptions of thinness and valence. Fourth, we did not collect data on the living circumstances of the Malaysian Chinese participants, but these participants were recruited in Selangor—a state with a high percentage urban population [82]. Research in Malaysia has found women in urban areas report lower body size preferences and greater body dissatisfaction than women in rural areas [18,42]; therefore, the results of this study may not apply to women in more rural areas of Malaysia. Fifth, some studies have found that the relationship between body dissatisfaction and attentional bias to thin bodies is potentially mediated by appearance comparisons and eating disorder specific rumination [31,73]. Evidence for this mediation is currently only correlational and not causal; however, future research could consider including these variables in their research design.

## 5. Conclusion

To the best of our knowledge, our study is the first to use a dot probe task to investigate the relationship between body dissatisfaction and attentional bias to thin bodies in both Western and non-Western women. We found no evidence of an association between body dissatisfaction and attentional bias to thin bodies. This lack of an association did not depend on the participant's ethnicity (White Australian versus Malaysian Chinese) or the ethnic congruence between participants and body stimuli used in the dot probe task (own-ethnicity versus other-ethnicity). Consistent with previous research [36,69,70], our dot probe task had low reliability. Free viewing eye-tracking paradigms are a more reliable measure of attentional bias [77,78] and have consistently produced evidence for a positive association between body dissatisfaction and attentional bias to thin bodies [7–11]. Therefore, it appears likely that our dot probe task was not reliable enough to detect this association. Thus, great caution must be applied before ruling out the possibility of group differences and own-ethnicity effects between White Australian and Malaysian Chinese women. Future research may employ eye-tracking techniques to investigate the moderating effects of ethnicity and ethnic congruency on the relationship between body dissatisfaction and attentional bias to body size.

## Data Availability

The study materials, analysis code and data for the participants recruited in Australia are publicly available from the University of Bristol Research Data Repository (DOI: 10.5523/bris.dn7ra6z7uats2asgp5k401te4 [83]; https://data.bris.ac.uk/data/dataset/dn7ra6z7uats2asgp5k401te4). For the participants recruited in Malaysia, we did not obtain explicit informed consent for data sharing. Therefore, we are unable to share the data for these participants due to ethical concerns. Instead, we have created a synthetic dataset for the Malaysian data which we have included in the data repository with the real Australian dataset. This synthetic dataset is clearly labelled and will enable interested researchers to reproduce our analyses on both datasets, even though the synthetic Malaysia dataset is not the real dataset. The editorial office has confirmed that the journal will make an exception to data sharing requirements on the basis that providing the dataset would be in violation of consent forms and present unacceptable ethical concerns. Supplementary material is available online [84].

## Footnotes

1 The splithalf package assumes attentional bias scores were calculated by subtracting mean reaction times on congruent trials from mean reaction times on incongruent trials. This is a simplified calculation compared to the attentional bias score used in our preregistered main analyses, because it involves two categories of trials (incongruent and congruent) rather than four (LPRT, LPLT, RPLT and RPRT). However, for the two methods of calculation the scores were almost perfectly correlated and our main analyses produced almost identical results for each (see electronic supplementary material).
